# Myelin Debris Stimulates NG2/CSPG4 Expression in Bone Marrow-Derived Macrophages in the Injured Spinal Cord

**DOI:** 10.3389/fncel.2021.651827

**Published:** 2021-03-19

**Authors:** Yang Liu, Grace Hammel, Minjun Shi, Zhijian Cheng, Sandra Zivkovic, Xiaoqi Wang, Pingyi Xu, Xijing He, Bing Guo, Yi Ren, Li Zuo

**Affiliations:** ^1^Department of Immunology, Guizhou Medical University, Guiyang, China; ^2^Department of Biomedical Sciences, Florida State University College of Medicine, Tallahassee, FL, United States; ^3^Department of Anesthesiology, The Affiliated Hospital of Guizhou Medical University, Guiyang, China; ^4^Department of Pathology, Guizhou Medical University, Guiyang, China; ^5^Department of Orthopedics, The Second Affiliated Hospital of Xian Jiaotong University, Xian, China; ^6^Department of Neurology, First Affiliated Hospital of Guangzhou Medical University, Guangzhou, China

**Keywords:** spinal cord injury (SCI), NG2, CSPG4, bone marrow derived macrophages, microglia, inflammation, myelin debris

## Abstract

Although the increased expression of members of the chondroitin sulfate proteoglycan family, such as neuron-glial antigen 2 (NG2), have been well documented after an injury to the spinal cord, a complete picture as to the cellular origins and function of this NG2 expression has yet to be made. Using a spinal cord injury (SCI) mouse model, we describe that some infiltrated bone marrow-derived macrophages (BMDMΦ) are early contributors to NG2/CSPG4 expression and secretion after SCI. We demonstrate for the first time that a lesion-related form of cellular debris generated from damaged myelin sheaths can increase NG2/CSPG4 expression in BMDMΦ, which then exhibit enhanced proliferation and decreased phagocytic capacity. These results suggest that BMDMΦ may play a much more nuanced role in secondary spinal cord injury than previously thought, including acting as early contributors to the NG2 component of the glial scar.

## Introduction

Spinal cord injury (SCI) is a devastating and potentially life-threatening form of injury that can not only cause life-long chronic pain and other injury-associated symptoms but also places an immense financial and social burden on the patients and their families. SCI can be divided into two defined phases: primary injury and secondary injury. Primary injury is characterized by the initial mechanical damage caused by the injury, which is closely followed by the multifaceted and chronic secondary injury. This secondary stage of SCI involves the propagation of an immense inflammatory cascade, widespread cell death, immune cell infiltration, disruption of the blood-spinal cord-barrier, and scar formation (Anwar et al., 2016). One key persistent characteristic of SCI that can occur as a result of the initial mechanical trauma, as well as develop as a progressive and chronic feature of secondary injury, is the demyelination of axons within and proximal to the injury site (Totoiu and Keirstead, 2005). It is this demyelination that leads to impaired axonal function and neuronal survival after injury (Almad et al., 2011). The myelin debris generated from demyelination is an important lesion-related factor that promotes injury progression. Myelin debris not only contains inhibitory molecules that prevent remyelination and axonal regeneration, but it also can activate an inflammatory response (McKerracher et al., 1994; Kotter et al., 2006; Sun et al., 2010; Geoffroy and Zheng, 2014; Lampron et al., 2015; Cantuti-Castelvetri et al., 2018; Kopper and Gensel, 2018). A critical cell type involved in this inflammatory response is BMDMΦ, which migrate to the injury area within 1–2 weeks post-injury (Fleming et al., 2006; Wang et al., 2015) and upon uptake of myelin debris not only become pro-inflammatory and persist at the lesion site but may also lose their normal functions such as a phagocytic capacity for apoptotic/necrotic cells and cellular debris. Together these changes may exacerbate the inflammatory microenvironment after injury (Fleming et al., 2006; Wang et al., 2015).

Neuron-glial antigen 2 (NG2), also known as chondroitin sulfate proteoglycan 4 (CSPG4), has been shown to inhibit axonal growth and its expression is highly upregulated after SCI, with expression levels increasing from 24 h after injury and peaking at 7 days (d) post-injury (Dou and Levine, 1994; Jones et al., 2002). NG2 positive cells accumulated within the glial scar have a significant proliferative capacity within days after injury, however, their function in glial scar formation, as well as their origin, remains poorly understood (Levine, 1994; Hackett and Lee, 2016). NG2 is not only present in oligodendrocyte precursor cells (OPCs) in the central nervous system (CNS), but also in pericytes, and activated macrophages and microglia (Bu et al., 2001; Jones et al., 2002; De Castro et al., 2005; Cejudo-Martin et al., 2016; Hesp et al., 2018). In several CNS cell types, NG2 has been characterized as an important factor in cell proliferation, migration, and survival (Burg et al., 1997, 1998; Stallcup, 2002; Stallcup and Huang, 2008; Kucharova and Stallcup, 2010). However, specifically regarding macrophages, NG2 has only been shown to play a role in their recruitment to brain tumors and areas of demyelination in the CNS (Cejudo-Martin et al., 2016; Stallcup et al., 2016). It is also important to note that NG2 expression is only induced in activated macrophages, however, in the context of SCI, the specific lesion related factor responsible for this activation and subsequent NG2 upregulation is as of yet unknown (Bu et al., 2001; Cejudo-Martin et al., 2016; Stallcup et al., 2016).

In the current study, we investigated the possible lesion-related factors that induce NG2/CSPG4 expression in BMDMΦ and microglia *in vitro* and in the injured spinal cord. We showed that NG2 expression is transiently colocalized with BMDMΦ mainly at the region of glial scar and that NG2 expression in BMDMΦ is upregulated upon myelin debris uptake* in vitro*. NG2^+^ BMDMΦ lose their phagocytic capacity and proliferate at a faster rate than NG2^−^ BMDMΦ. These data suggest that increased NG2 expression in BMDMΦ in the injury perimeter may be due to the uptake of myelin debris—an abundant lesion-related factor generated immediately after an injury. Finally, we demonstrated that myelin debris engulfment-induced NG2 expression is associated with an alteration of BMDMΦ functions such as phagocytic and proliferative capacity.

## Materials and Methods

### Reagents

All chemicals were purchased from Sigma–Aldrich unless otherwise noted here. Dulbecco’s Modified Eagle Medium (DMEM) with high glucose was purchased from Hyclone (SH30003.03), and all other cell culture media was purchased from Invitrogen (Carlsbad, CA, USA) unless otherwise indicated. Recombinant mouse TGF-β1 (#5231) was from Cell Signaling Technology. Anti-MBP (ab40390; 1:200 for IF) was purchased from Abcam. GAPDH [ab181602; 1:3,000 for western blot (WB)] was purchased from Abcam. F4/80 antibody was produced *via* hybridoma cell line HB-198. All cell lines were purchased from American Tissue Culture Collection (ATCC, Manassas, VA, USA). Alexa Fluor 555- and 647-conjugated secondary antibodies (1:1,000 for IF) were purchased from Invitrogen.

### Mice

C57BL/6J (wild type, WT), C57BL/6-Tg(CAG-EGFP)^1Osb/J^ (EGFP), B6.129P2(Cg)-Cx3cr1^tm1Litt/J^ (CX3CR1^GFP/+^), and B6.Cg-Tg(CAG-mRFP1)^1F1Hadj/J^ (RFP) mice were purchased from Jackson Laboratory (Bar Harbor, ME, USA) and maintained in a pathogen-free animal facility in Florida State University. Animal housing, handling, and all procedures were approved by the Animal Care and Use Committee (ACUC) of Florida State University. All results from animal work were evaluated and quantified using double-blinded methods.

### Spinal Cord Injury in Mice

Thoracic spinal cord contusion injuries were performed on 8–12 weeks-old female mice. To expose the spinal cord, a laminectomy was performed on the T8–T11 vertebrae. The contusion injury was induced using the NYU impactor with a 5 g rod dropped 6.25 mm from the cord surface as described in our previous study (Zhou et al., 2019).

### Generation of GFP^+^ and RFP^+^ Mouse Bone Marrow Chimeras

GFP^+^ and RFP^+^ bone marrow chimeric mice were generated according to a previous publication (Wang et al., 2015). C57BL/6 WT mice at 6–8 weeks of age were anesthetized using ketamine/xylazine and exposed to a single dose of irradiation with a maximum of 12 Gy and a maximum dosage rate of 4.54 Gy/min using an X-RAD 320 self-contained irradiation system (Precision X-Ray, North Branford, CT, USA). The brain and spine were protected from exposure. Following irradiation, mice received either PBS or 5 × 10^6^ bone marrow cells freshly collected from transgenic GFP or RFP mice intravenously (*i.v*.). Efficient reconstitution was confirmed by postmortem examination of circulating blood for GFP^+^ or RFP^+^ cells. On average, 80% transplant engraftment efficiency was achieved.

### Cell Culture

BMDMΦ were prepared as previously described (Wang et al., 2012, 2015). Bone marrow cells from mice up to 12 weeks of age were collected by flushing the femoral shafts with DMEM supplemented with 5% newborn calf serum (NCS). Cells were then cultured in an incubator at 37°C, with 5% CO_2_, for 7 days in DMEM supplemented with both 5% NCS and 15% L929 conditioned media. Mouse microglia cell lines (BV2 cells, Accegen, Cat: ABC-TC212S) were cultured in DMDM supplemented with 10% FBS.

### Myelin Debris Preparation

Myelin debris was isolated and prepared as previously described and utilized at a concentration of 100 mg/ml in each experiment (Sun et al., 2010). Brains from WT mice are isolated and homogenized and then processed through density gradient centrifugation in which the homogenized brains are diluted in a 0.32 M sucrose solution and then added to a 0.83 M sucrose solution and spun down through ultracentrifugation to produce a crude myelin lysate intermediate layer. This crude lysate is then washed with Tris-Cl buffer and spun down using ultracentrifugation several times resulting in a highly purified myelin debris fraction.

### Phagocytosis Assay

BMDMΦ were washed twice with PBS then treated for 3 h with a final concentration of 1 mg/ml of myelin debris diluted in DMEM. The cells were then washed with PBS to remove any non-ingested myelin debris before preceding with immunocytochemistry staining.

### Histology and Immunofluorescent Staining

Mice were transcardially perfused with 0.9% saline and then 4% paraformaldehyde (PFA). Spinal cords were collected and fixed in 4% PFA overnight, and then 30% sucrose at 4°C. Cords were then frozen and mounted in optimal cutting temperature compound before being sectioned. The sectioned cords were then washed with PBS and blocked with PBS containing 0.3% Triton X-100 and 1% BSA for 1 h at room temperature. Then samples were incubated overnight with their respective primary antibodies and then washed and incubated with secondary antibodies for 1.5 h at room temperature. Regions of interest were imaged with a Nikon Ti-E microscope and Nikon A1 laser scanning confocal microscope (Nikon Instruments, Melville, NY, USA). All images were taken from sagittal sections of the injury area. Using Nikon NIS-Elements software, the regions of the spinal cord were outlined and measured at 200 μm intervals over a 2 mm distance, centered on the lesion core. The injured regions were defined as the regions with a radius of around 300 μm, which were negative/weak positive for NG2 but densely positive for nucleus nuclear staining (Hoechst staining). The marginal regions, within the NG2^+^ glial scar, were considered as 300–600 μm away from the epicenter. At least 200 cells were quantified per image per area per mouse. The images were processed using ImageJ2 and the ImageJ distribution Fiji (Schindelin et al., 2012; Rueden et al., 2017).

### Quantitative RT-PCR

Total RNA from cells was isolated using TRIzol and reverse transcribed into cDNA according to manufacturer’s instructions using High Capacity cDNA Reverse Transcription Kit (#4368814; Applied Biosystems). cDNA was treated with DNase (RQ1 RNase-Free DNase, cat: M6101PCR) and qPCR was performed according to manufacturer’s instructions using PowerUp SYBR Green Master Mix (ref:100029284). All reactions (20 ul) were run using a real-time PCR system (CFX96; BioRad), and the specificity of every primer was determined using melting curve analysis. The expression level of target genes was normalized to *18S* and calculated using ΔΔ^Ct^. Primers: *NG2/CSPG4*: Forward 5′-TTCCTTCGCCTTACAAGTCC-3′, Reverse 5′-CTCACTCACCAGGAGCTGTAG-3′. *18S*: Forward 5′-CTCTTAGCTGAGTGTCCCGC-3′, Reverse 5′-CTGATCGTCTTCGAACCTCC-3′.

### Enzyme-Linked Immunosorbent Assay (ELISA) Detection of CSPG4

CSPG4 protein in BMDMΦ was quantified by a commercial quantitative sandwich ELISA kit (G-Biosciences Cat. #IT5412). All samples were normalized to total protein. Data are reported as pg of CSPG4/mg total protein.

### Statistical Analysis

Data distribution was assumed to be normal, but this was not formally tested. The statistical significance between control and experimental groups was determined using independent *t*-tests, using Prism 7 (GraphPad). Differences were considered statistically significant when *P* ≤0.05. With *P ≤0.05, **P ≤0.01, ***P ≤ 0.001, and ****P ≤ 0.0001 as shown in figures and figure legends unless otherwise denoted. Data are shown as mean ± SD unless otherwise indicated.

## Results

### BMDMΦ Express NG2 in the Lesion Perimeter at 1-Week Post-SCI

**Figure 1:**
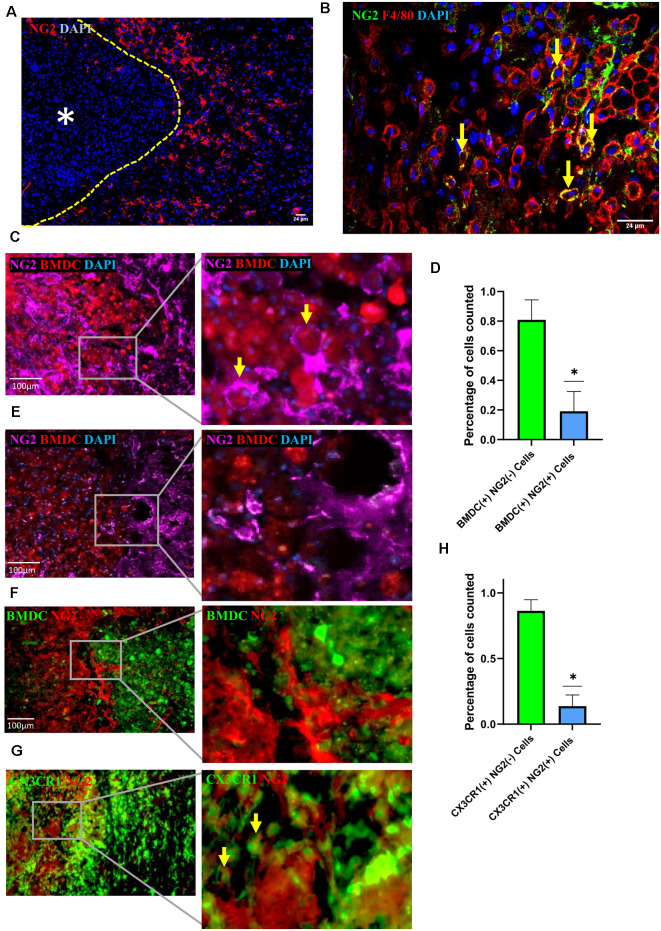
Neuron-glial antigen 2 (NG2) expression in injured spinal cords. **(A)** Representative image of NG2 expression (red) in the marginal regions of the injured spinal cord at 2 week post-injury with yellow dashed line demarcating lesion edge and asterisk the lesion core. **(B)** NG2 (green) colocalization with F4/80 (red) positive cells within the marginal region of the injured cord at 1 week post-injury. **(C–E)** Representative image showing colocalization between RFP^+^ bone marrow-derived cells (BMDCs) and NG2 (purple) within the marginal region at 1 week **(C)** and 2 week **(E)** post-injury in a cord from an RFP bone marrow (BM) chimeric, with yellow arrows highlighting two NG2^+^ BMDCs. **(D)** Quantification of RFP^+^ BMDCs with or without NG2 colocalization within the marginal region 1 week post-injury; data are shown as means ± SD (*n* = 4), with at least three images quantified per mouse from four separate mice. *P ≤ 0.05. **(F)** Representative image showing colocalization between GFP^+^ BMDCs and NG2 (red) within the marginal region in a 2 week post-injury cord from a GFP bone marrow chimeric mouse; data are shown as means ± SD (*n* = 4), with at least three images quantified per mouse from four separate mice. *P ≤ 0.05. **(G)** Representative image showing colocalization between CX3CR1^+++^ (green) and NG2 (red) within the marginal region at 10 days post-injury cord from CX3CR1^GFP/+^ mouse. The yellow arrows highlighting two microglia expressing NG2. **(H)** Quantification of CX3CR1^+++^ microglia with and without NG2 colocalization within the marginal region; data are shown as means ± SD (*n* = 3), with at least three images quantified per mouse from three separate mice. *P ≤ 0.05.

We, as well as others, previously reported that BMDMΦ accumulate in the lesion perimeter during the first 1–2 weeks (w) after injury (Fleming et al., 2006; Wang et al., 2015). Within days after SCI, the lesion core begins to be surrounded by the glial scar, which is formed by a highly proliferative population of reactive astrocytes and microglia (Hu et al., 2010; Yuan and He, 2013; Adams and Gallo, 2018; Alizadeh et al., 2019; Yang et al., 2020). Consistent with previous studies, we found that NG2 positive cells were detected in the marginal region of the lesion core in SCI mice (Figure 1A), with NG2 expression found to be 10 times greater in mice with a demyelinating injury vs. control mice (Novotna et al., 2011; Hackett et al., 2016; Levine, 2016; Kucharova and Stallcup, 2017; Hesp et al., 2018). It has been well established that NG2 is not only expressed by OPCs and pericytes, but also by activated macrophages and microglia (Bu et al., 2001; De Castro et al., 2005; Zhu et al., 2012; Cejudo-Martin et al., 2016; Hesp et al., 2018). Here we examined the spatiotemporal relationship between cell types present at the injury site and NG2 expression in injured spinal cords. At 1 week post-injury, we observed a proportion of F4/80 positive cells expressing NG2 (Figure 1B), leading us to further investigate macrophages and microglia as other sources of NG2 after injury. To determine if these F4/80 positive cells are resident microglia or infiltrated BMDMΦ, we generated a bone marrow (BM) chimeric mouse model, in which the bone marrow-derived cells (BMDCs) of WT recipient mice were replaced by donor BMDCs isolated from RFP mice. Using this model, we observed colocalization between a small proportion of infiltrated BMDCs and NG2 at 1 week after injury (Figures 1C,D). Utilizing the same model, our previous study demonstrated that 1 week after SCI, the BMDCs around the lesion epicenter were BMDMΦ since these BMDCs colocalized with key macrophage markers such as F4/80, CD68, and IBA-1, indicating that a vast majority of these cells were BMDMΦ, rather than locally activated microglia (Wang et al., 2015). Therefore, these NG2^+^ BMDCs at the injury site are considered as infiltrated BMDMΦ. In the same lesion perimeter area 2 week after injury, a vast majority of BMDMΦ migrated into the injury core, and in these BMDMΦ, as well as those not yet migrated into the core, very few showed colocalization with NG2 (Figure 1E). These results were further confirmed in a GFP BM chimeric mouse model where again, 2 week after injury, BMDMΦ lacked expression of NG2 (Figure 1F). In our previous study, utilizing a CX3CR1^GFP/+^ transgenic mouse model, we demonstrated that strong CX3CR1 expression can only be seen in microglia after injury making it a useful tool in identifying microglia in the injured cord (Wang et al., 2015). We, therefore, utilized the CX3CR1^GFP/+^ mouse model in our current study to further elucidate the identity of NG2 expressing cells at the injury site. There did appear to be some colocalization of CX3CR1 and NG2 at 10 days after injury, however, the quantification of NG2 expression in the CX3CR1 strong positive populations demonstrated that there were fewer NG2^+^ microglia than NG2^+^ BMDMΦ (Figures 1G,H). This data together suggests that BMDMΦ, as well as microglia, contributes to the increase in NG2 expression near the marginal area of the injury site approximately 1 week after injury.

### *In vitro* Myelin Debris Engulfment Causes Upregulation of NG2 Expression in BMDMΦ

**Figure 2:**
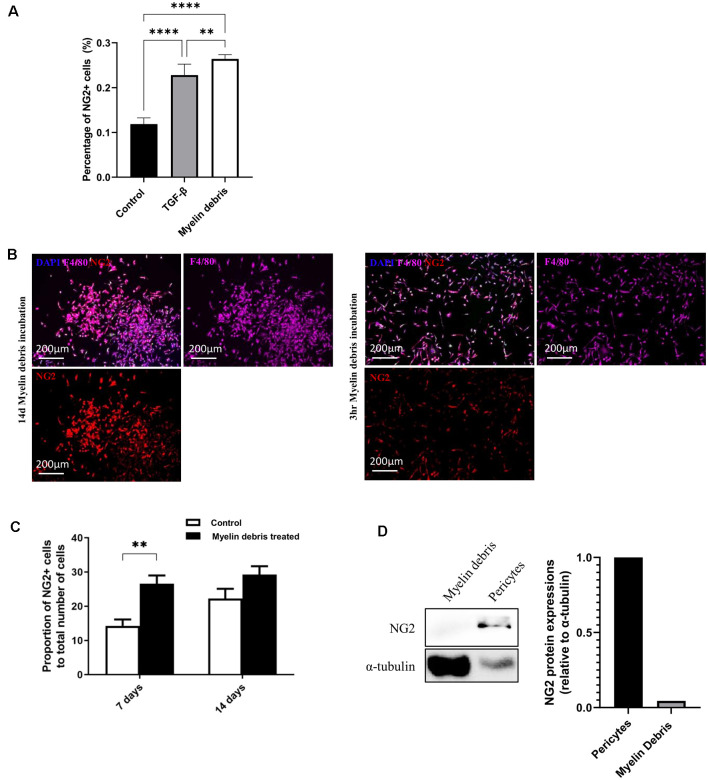
Myelin debris engulfment stimulates NG2 expression in BMDMΦ. **(A)** Percentage of NG2 expressing BMDMΦ after treatment with myelin debris or TGF-β for 14 days. **(B)** Representative images of NG2 expression (red) in BMDMΦ (F4/80, purple) cultured and treated with myelin debris for 14 days (left) or cultured for 14 days and then treated with myelin debris for 3 h (right). **(C)** The proportion of NG2 expressing BMDMΦ treated with myelin debris for 7 days and 14 days, respectively. Data for all quantifications are shown as means ± SD of three separate BMDMΦ isolations from three separate mice (*n* = 3). **P ≤ 0.01 and ****P ≤ 0.0001. **(D)** NG2 protein in myelin debris and mouse primary pericytes detected by Western blot (WB) images. Corresponding quantification of protein levels was determined by densitometry analysis relative to α-tubulin.

With our previous results showing a large number of NG2^+^ cells surrounding the injury area, we hypothesize that lesion-related factors could be contributing to NG2 expression. One potential injury factor is the myelin debris generated from myelin sheaths of axons that are damaged upon injury. Myelin debris can be detected within 24 h after injury, increases over a 1 week period, and accumulates in the lesion site for months or even years after injury (Buss et al., 2005; Kopper and Gensel, 2018). Microglia and BMDMΦ are classified as the two major professional phagocytes that engulf and clear myelin debris in the injury area (Rotshenker, 2003; Kopper and Gensel, 2018). We, therefore, investigated the potential role of myelin debris as a lesion-related factor responsible for the induction of NG2 expression in these two types of phagocytes. BMDMΦ was treated with myelin debris for 14 days. The expression of NG2 in BMDMΦ was markedly increased compared with control BMDMΦ (without myelin debris treatment; Figure 2B). Furthermore, the NG2^+^ cells also express F4/80 (Figure 2B), highlighting the fact that these NG2^+^ cells are indeed macrophages. Notably, myelin debris possessed a significantly greater influence on NG2 expression in BMDMΦ than TGF-β, a known inducer of NG2 expression/secretion in macrophages (Moransard et al., 2011; Figure 2A). We next performed a similar experiment observing NG2 expression in BMDMΦ over a series of time points of myelin debris treatments. After 3 h, 1 day, 2 days, and 3 days we did not observe a significant increase in NG2 expression in BMDMΦ (data not shown). However, after 7 days of myelin debris treatment, BMDMΦ showed a very significant increase in NG2 expression and a small increase after 14 days of myelin debris treatment (Figure 2C). These results parallel our *in vivo* observations where BMDMΦ only showed a significant increase in NG2 expression after 1 week of myelin debris exposure. To rule out myelin debris as a potential contributor of NG2 protein in our BMDMΦ cultures, we performed a western blot analysis of myelin debris and pericyte cells, a known NG2 expressing cell. The results showed that myelin debris contains virtually no NG2 protein and therefore it is unlikely myelin debris is a contributor to NG2 expression changes in BMDMΦ (Figure 2D). We also observed that macrophages that have taken-up myelin debris for less than 3 days do not display any NG2 expression, further demonstrating that myelin debris treatment in itself does not introduce NG2 protein into our cell cultures (data not shown).

**Figure 3:**
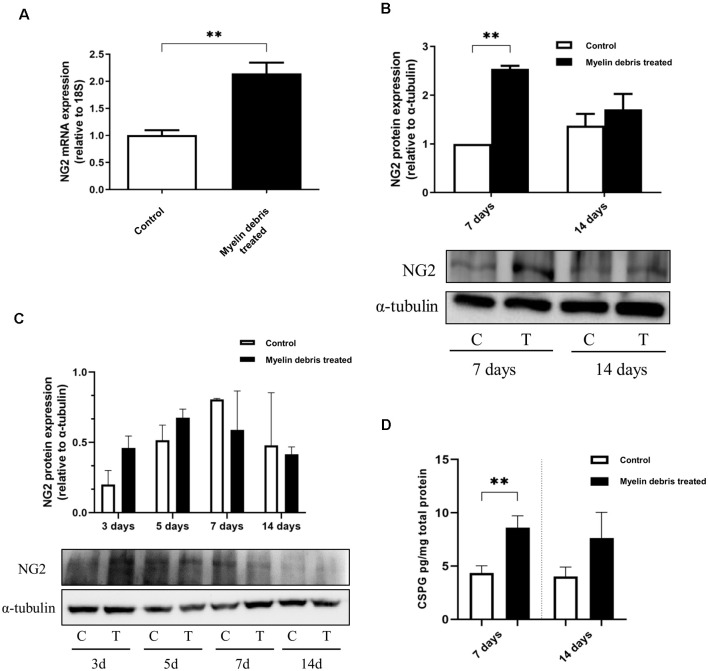
NG2 expression at levels of the mRNA, intracellular protein, and secreted protein in BMDMΦ treated with myelin debris. **(A)** NG2 mRNA expression in BMDMΦ treated with myelin debris for 7 days. **(B)** NG2 protein expression in BMDMΦ treated with myelin debris for 7 days and 14 days assessed by WB images. Corresponding quantification of protein levels was determined by densitometry analysis relative to α-tubulin. The immunoblots were performed twice with similar results. C: control; T: myelin debris treatment. **(C)** NG2 protein expression in microglia with myelin debris for indicated time points assessed by WB images. Corresponding quantification of protein levels was determined by densitometry analysis relative to α-tubulin. The immunoblots were performed four times with similar results. C: control; T: myelin debris treatment. **(D)** Chondroitin sulfate proteoglycan 4 (CSPG4) in the supernatant of BMDMΦ treated with myelin debris for 7 days and 14 days detected by Enzyme-linked immunosorbent assay (ELISA). Data for all quantifications are shown as means ± SD of three separate BMDMΦ isolations from three separate mice (*n* = 3). **P ≤ 0.01.

To further confirm the ability of myelin debris to induce NG2 expression in BMDMΦ, we investigated NG2 at the levels of mRNA, intracellular protein, and secreted protein in BMDMΦ treated with myelin debris. NG2 mRNA level was significantly higher in myelin debris treated cells after 7 days (Figure 3A). In a similar temporal pattern to BMDMΦ in the injury site after injury, BMDMΦ treated with myelin debris for 7 days, rather than for 14 days, showed significantly increased expression of NG2 protein (Figure 3B). BV2 microglia treated with myelin debris for the indicated time points did show a slight increase in NG2 expression only at 3d of treatment, however, the change is not statistically significant (Figure 3C). Moreover, CSPG4, the secreted form of NG2, also showed significantly higher levels in the conditioned media of BMDMΦ treated with myelin debris for 7 days and 14 days (Figure 3D). These data firmly suggest that myelin debris is an SCI lesion-related factor capable of inducing NG2/CSPG4 expression in BMDMΦ.

### NG2 Expression in BMDMΦ Is Associated With Increased Proliferation and Impaired Phagocytic Capacity

**Figure 4:**
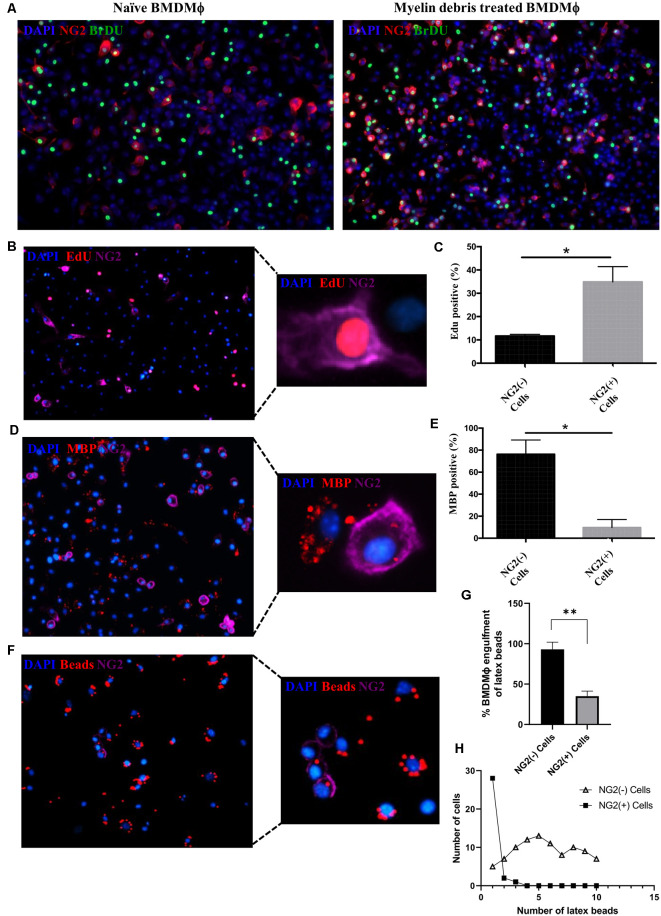
Alteration of proliferation and phagocytic capacity in NG2^+^ BMDMΦ. **(A)** Representative image of NG2 expression (red) and proliferation marker BrdU (green) in naïve BMDMΦ (absence of myelin debris; left) and BMDMΦ treated myelin debris for 14 days (right). **(B)** Representative image of EdU^+^ (red) with NG2^+^ (purple) BMDMΦ treated myelin debris for 14 days; inset showing detailed immunostaining of a single EdU^+^/NG2^+^ BMDMΦ. **(C)** Percentage of EdU^+^ in NG2^+^ BMDMΦ and NG2^−^ BMDMΦ. **(D)** Representative image of BMDMΦ phagocytosis of myelin debris in NG2^+^ BMDMΦ and NG2^−^ BMDMΦ. Engulfed myelin debris was determined by detection of intracellular myelin basic protein (MBP; red) puncta; insert showing detailed immunostainings of NG2^+^ BMDMΦ and NG2^−^ BMDMΦ containing MBP puncta. **(E)** The corresponding percentage of NG2^+^ BMDMΦ and NG2^−^ BMDMΦ phagocytosis of myelin debris. **(F)** Representative image of BMDMΦ phagocytosis of latex beads (red) in NG2^+^ BMDMΦ and NG2^−^ BMDMΦ; insert showing detailed immunostaining of NG2^+^ BMDMΦ and NG2^−^ BMDMΦ containing latex beads. **(G)** The corresponding percentage of latex beads engulfment in NG2^+^ BMDMΦ and NG2^−^ BMDMΦ. **(H)** The number of engulfed beads in both NG2^+^ BMDMΦ and NG2^−^ BMDMΦ. Data are shown as means ± SD of three separate BMDMΦ isolations from three separate mice (*n* = 3). *P ≤ 0.05 and **P ≤ 0.01.

We next investigated any functional changes occurring in NG2^+^ BMDMΦ treated with myelin debris. After myelin debris treatment for 14 days, NG2^+^ BMDMΦ showed higher proliferative ability than NG2^−^ BMDMΦ as indicated by higher levels of BrdU and EdU (Figures 4A–C). A previous study demonstrated that in response to LPS stimulation in the rat brain, NG2 expression was induced in macrophages and microglia and they lost their phagocytic capacity for latex beads (Zhu et al., 2012). Therefore, we next investigated if myelin debris-induced NG2 expression could be associated with BMDMΦ loss of phagocytic capacity for myelin debris and latex beads. BMDMΦ were treated with myelin debris for 7 days and a significantly higher proportion of NG2^−^ BMDMΦ possessed the ability to phagocytose myelin debris compared to NG2^+^ BMDMΦ, as seen by the intracellular myelin basic protein (MBP) puncta (Figures 4D,E). Similarly, upon treatment with latex beads, more NG2^−^ BMDMΦ phagocytosed the latex beads compared to NG2^+^ BMDMΦ (Figures 4F,G). Beyond that, NG2^−^ BMDMΦ were able to take up more latex beads than NG2^+^ BMDMΦ, with a vast majority of NG2^+^ BMDMΦ engulfing only one bead (Figure 4H). Together this evidence suggests that NG2 expression is associated with impaired BMDMΦ phagocytic capacity.

## Discussion

Although it has been reported that NG2 expression can be found in microglia and macrophages, little is known specifically regarding the consequences of NG2 expression in infiltrated macrophages at the injury site of SCI, as well as what environmental factors contribute to this expression (Bu et al., 2001; De Castro et al., 2005; Moransard et al., 2011; Cejudo-Martin et al., 2016; Hesp et al., 2018). Our data demonstrate that BMDMΦ can express NG2 approximately 1 week after SCI in the lesion perimeter. We revealed that myelin debris is a lesion-related factor capable of stimulating NG2/CSPG4 expression and secretion in BMDMΦ *in vitro*. NG2 expression can also be observed in BMDMΦ and microglia in the injured spinal cord in a similar temporal manner. Furthermore, we uncovered that these NG2 expressing BMDMΦ possess impaired phagocytic capacity and increased proliferative ability.

NG2/CSPG4 is a membrane-spanning protein with intracellular and extracellular domains that facilitate NG2 signaling. Differential phosphorylation of two sites on the intracellular domain of NG2, and β1 integrin’s interaction with these different forms of phosphorylated NG2, are key mediators of cell motility and cell proliferation (Fukushi et al., 2004; Makagiansar et al., 2007; Binamé et al., 2013; Yadavilli et al., 2016). In the context of infiltrated macrophages expressing NG2 in the CNS, the mechanism by which NG2 expression is induced, as well as the function of NG2 in these cells has yet to be completely elucidated. It has been reported that NG2^+^ BMDMΦ in the ischemic lesions of the human and rat brain plays a beneficial role, at least in part, by their production of neuroprotective factors (Smirkin et al., 2010). Furthermore, NG2 appears to play a crucial role in macrophage migration since there was a significant decrease in macrophage abundance in brain tumors and sites of CNS demyelination in myeloid-specific NG2 null mice (mye-NG2-KO mice; De Castro et al., 2005; Cejudo-Martin et al., 2016; Kucharova and Stallcup, 2017). In a lysolecithin-induced spinal cord demyelination mouse model, the lesion repair in both mye-NG2-KO mice and NG2 null mice is greatly reduced compared with WT mice (Kucharova and Stallcup, 2015; Cejudo-Martin et al., 2016). The substantial decrease in repair in these mice may simply be the result of the greatly decreased presence of macrophages at the demyelinated sites, which would then lead to decreased clearance of debris. The loss of NG2 expression leads to decreased proliferation of OPCs, pericytes, and macrophages/microglia, and without NG2 there is a shift from pro-inflammatory to anti-inflammatory cytokine production in macrophages and microglia (Kucharova et al., 2011). This shift in cytokine production brings about many important questions as to the role NG2 could be playing in SCI repair through the mediation of BMDMΦ inflammatory profile and deserves further investigation.

Most of what is known about NG2 in the context of SCI is focused on its expression in pericytes within the lesion core and OPCs in the lesion border and glial scar. However, few studies have placed attention on macrophages and microglia as NG2 expressing cells or their role in SCI progression and repair. A population of NG2 expressing cells has been shown to proliferate within days after injury and accumulate within the glial scar, however, their function in glial scar formation, as well as their origin, remains poorly understood (Levine, 1994; Hackett and Lee, 2016). The glial scar is a dense barrier of scar tissue generated by a highly proliferative population of reactive astrocytes and NG2 positive cells, serving as both a protective and inhibitory structure for healing (McTigue et al., 2001; Jones et al., 2002; Hu et al., 2010; Yuan and He, 2013; Adams and Gallo, 2018; Yang et al., 2020). The glial scar not only acts as a physical barrier to axonal regrowth but also contains neurotoxic reactive astrocytes, as well as NG2 and its secreted form CSPG4 which can act as inhibitory molecules for axonal growth (Dou and Levine, 1994; Schäfer and Tegeder, 2018; Yang et al., 2020). It is not very clear if NG2 expressing BMDMΦ have a beneficial or detrimental role in secondary injury after SCI. With the knowledge that NG2/CSPG4 are major contributors to the inhibitory nature of the glial scar, gaining a further understanding of the role that these CSPG4 producing NG2^+^ BMDMΦ play in the deposition of CSPGs in the glial scar is of great importance.

We have previously shown that myelin debris engulfment polarized BMDMΦ toward a chronic pro-inflammatory M1 phenotype (Wang et al., 2015), and with this current study we believe it is reasonable to infer that these pro-inflammatory cells are probably NG2^−^ BMDMΦ as NG2^+^ BMDMΦ have a poor phagocytic capacity. This therefore could be evidence of NG2^+^ BMDMΦ playing a beneficial role in reducing inflammation at the injury site, however, future studies are needed to separate and define the inflammatory features of NG2^+^ BMDMΦ and NG2^−^ BMDMΦ in response to myelin debris. The lower phagocytic capacity shown in these NG2^+^ BMDMΦ could also prove detrimental by hindering clearance of myelin debris which accumulates at the injury site. Further investigation is required to understand if NG2^+^ BMDMΦ also possess a lower phagocytic capacity for other damaged cells/tissue at the lesion site.

**Figure 5:**
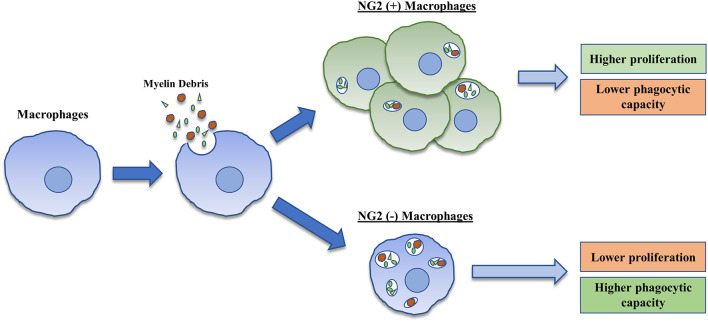
Graphical representation of the induction and consequences associated with NG2 expression in BMDMΦ. BMDMΦ upon treatment with myelin debris demonstrate an increase in expression and secretion of NG2/CSPG4 in a small percentage of their cell population. These NG2 positive cells demonstrate increased proliferation and possess a lower phagocytic capacity than NG2 negative BMDMΦ.

This study implicates that an SCI lesion-related factor may be responsible for the induction of NG2 expression in BMDMΦ and that this expression of NG2 is associated with several different functionality changes in BMDMΦ including increased proliferation and reduced phagocytic capacity (Figure 5). Overall, NG2 expression in BMDMΦ merits great interest in the context of secondary SCI, and future studies are needed to understand the precise roles of NG2 on BMDMΦ function in the injured spinal cord. These investigations could prove to be invaluable for the development of different therapeutic strategies targeting components of the secondary injury cascade after SCI.

## Data Availability Statement

The raw data supporting the conclusions of this article will be made available by the authors, without undue reservation.

## Ethics Statement

The animal study was reviewed and approved by Florida State University’s Animal Care and Use Committee.

## Author Contributions

YR and LZ conceived and designed the study. YL, GH, MS, ZC, SZ and XW performed experiments and analyzed the data. PX, XH, BG and LZ contributed to discussion. GH and YR wrote the manuscript. All authors contributed to the article and approved the submitted version.

## Conflict of Interest

The authors declare that the research was conducted in the absence of any commercial or financial relationships that could be construed as a potential conflict of interest.
